# Discrimination of Borderline Personality Disorder (BPD) and Attention-Deficit/Hyperactivity Disorder (ADHD) in adolescents: Spanish version of the Borderline Personality Features Scale for Children-11 Self-Report (BPFSC-11) Preliminary results

**DOI:** 10.1186/s40479-023-00223-2

**Published:** 2023-05-16

**Authors:** Natalia Calvo, Jorge Lugo Marin, Raquel Vidal, Carla Sharp, Juan D. Duque, Josep-Antoni Ramos-Quiroga, Marc Ferrer

**Affiliations:** 1 Psychiatry Department, Centro de Investigación Biomédica en Red de Salud Mental (CIBERSAM), Hospital Universitari Vall d’HebronUniversitat Autònoma de Barcelona, Passeig Vall d´Hebron 119-129, 08035 Barcelona, Spain; 2 Psychiatry, Mental Health and Addictions Group. Vall d’Hebron Institut de Recerca (VHIR), Barcelona, Spain; 3 Psychiatry and Legal Medicine Department, Universitat Autònoma de Barcelona (UAB), Barcelona, Spain; 4 Grup TLP Barcelona (Grup TLP BCN), Barcelona, Spain; 5 Department of Psychology, University of Houston, Texas, USA

**Keywords:** Borderline Personality Features Scale for Children-11 Self Report (BPFSC-11), Borderline Personality Disorder (BPD), Attention-Deficit/Hyperactivity Disorder (ADHD), Adolescence, Discriminate Capacity

## Abstract

**Background:**

Although the diagnosis of Borderline Personality Disorder (BPD) during adolescence has been questioned, many recent studies have confirmed its validity. However, some clinical manifestations of BPD could be identifiable in adolescents with other pathologies, such as Attention-Deficit/Hyperactivity Disorder (ADHD). The objective of the present study is to examine the capacity of the self-report Borderline Personality Features Scale Children-11 (BPFSC-11) to discriminate between BPD and ADHD adolescents.

**Methods:**

One hundred and forty-five participants were grouped based on their diagnosis: 58 with BPD, 58 with ADHD, and 29 healthy volunteers as a control group. Between-group differences and the ROC curve were performed to test if the total score for the BPFSC-11 and/or its factors can significantly discriminate between BPD and other adolescent groups.

**Results:**

The results show that the total BPFSC-11 score has good discriminant capacity among adolescents diagnosed with BPD, ADHD and healthy volunteers. However, different patterns of discriminative capacity were observed between the three groups for emotional dysregulation and impulsivity/recklessness factors.

**Conclusions:**

Our results support the hypothesis that the BPFSC-11 is an adequate instrument for discriminating between BPD and ADHD in adolescents, who can present significant psychopathological overlap. Tools to identify BPD in adolescence, as well as for better differential diagnosis, would improve the possibility of offering specific treatments targeting these populations.

## Background

Borderline Personality Disorder (BPD) is a severe mental disorder characterized by a pervasive pattern of instability in affect regulation, pronounced impulsive and self-damaging behavior, unstable identity and difficulties in emotion regulation [1]. Although historically the diagnosis of BPD in adolescence has been long debated, in recent years there has been increased evidence to suggest that BPD symptoms are present in childhood and adolescence, and that BPD can be reliably diagnosed in adolescence [2–6]. In fact, Miller et al. (2008) had already pointed to the existence of a body of evidence indicating that BPD is reliably diagnosed in adolescence and its reliability and validity is comparable with adults [6]. In the last two decades, there has been greater sensitivity of diagnosis which is legitimated in the psychiatric nomenclature (DSM-5 and DMS-5-TR) [1, 7].

It is recognized that BPD in adolescence contributes to “adaptive failure” [8] and that it has a strong impact, with negative outcomes in different areas of functioning and serious repercussions for psychopathological aspects, as well as in educational, occupational, social, and legal settings, and a high economic cost for the healthcare system [9–11]. Although prevalence studies in adolescents are still scarce, the information published to date indicates ranges of 1–3% for the general adolescent population, with cumulative prevalence rates of 1.4% by 16 years old, rising to 3.2% by the age of 22 [4, 12–16]. In mental health settings, this disorder can be found in about 11% of outpatients and in as many as 50% of inpatients (between 33% and 43–49% according to studies) [4, 12–16]. Cumulative evidence also suggests that BPD adolescents can benefit from early intervention when they are properly diagnosed [4, 10]. However, other recent studies have shown that there is a long way to go before this evidence is introduced in routine clinical practice [12]. Unfortunately, diagnosis and consequent therapeutic intervention are often delayed, with adverse results for the patients, involving a high risk of progression to impairment, more severe psychiatric pathology and comorbidity, and poorer clinical and psychosocial functioning in early adulthood, among others [15].

Identification of adolescents with BPD is still difficult. There is a remarkable clinical overlap between BPD and other psychopathological disorders that begin in childhood or adolescence, such as attention-deficit/hyperactivity disorder (ADHD) and clinicians express concern over the distinction between BPD and ADHD [2]. ADHD is a highly prevalent disorder in adolescence, and impulsivity and emotional instability, classically considered core symptoms of BPD, are part of its clinical characteristics [17, 18]. Studies report that BPD and ADHD are commonly comorbid in both adults and adolescents [12]. Philipsen et al. [19] found that 41% of adults with BPD reported high rates of ADHD in childhood, and the authors have reported that severe BPD symptoms in adulthood were related to a diagnosis of ADHD in childhood. More specifically, Miller et al. [20] reported that 13.5% of children with ADHD, but only 1.2% of the control group, were diagnosed with BPD in adolescence. These authors also reported that youth who continued to meet criteria for ADHD diagnosis in adolescence had higher rates of BPD compared to youth who remitted from ADHD. As a result, research into the relationship between BPD and ADHD has increased, and data has even been presented on possible etiopathogenic bases that are common to both disorders [2, 12, 19, 21].

Although BPD and ADHD have common aspects, accurate diagnosis can be achieved considering the core dysfunctional areas of both conditions [15], as well as differences related to the therapeutic strategies indicated for each, so it is essential to make a correct differential diagnosis between the two disorders to ensure a subsequent comprehensive and specific approach to each one. In this way, while adolescents can benefit from psychotherapeutic intervention programs that are specially designed and adapted for BPD, such as Dialectical Behavior Therapy-Adolescents (DBT-A), Emotion Regulation Training (ERT), Mentalization-Based Therapy-Adolescents (MBT-A) and/or a combination of psychodynamic and cognitive behavioral therapy such as Cognitive Analytic Therapy (CAT) [22, 23], the clinical guides recommend multi-modal programs for ADHD patients which combine pharmacological with psychosocial treatment [24, 25].

While there are a significant number of validated instruments for the diagnosis of ADHD [22], these are still scarce for BPD diagnosis in adolescents. The Borderline Personality Features Scale for Children-11 (BPFSC-11) [26] screening instrument has been specifically designed for BPD and uses self-reporting to evaluate borderline personality, as well as the subclinical levels of this pathology, in children from 9 years of age to adolescents. It is based on two previous instruments: the Personality Assessment Inventory Borderline Subscale (PAI-BOR) [27] and the Borderline Personality Features Scale for Children (BPFSC) [3]. After examining the structure of the latter through a multidimensional confirmatory item response theory (IRT), its authors [26] proposed the current 11-item version, which has been shown to have adequate psychometric properties with regard to validity (concurrent and criterion) and reliability for the screening of BPD in the adolescent population [28].

The objective of this study is to test the capacity of the Spanish version of the BPFSC-11 to discriminate between BPD and ADHD in adolescents. Its psychometric properties of reliability, validity, sensitivity and specificity are also preliminarily analyzed.

## Methods

### Participants

Participants in the current sample were recruited between October 2018 and February 2020 from the outpatient section of Psychiatry Department of a university teaching hospital in Barcelona, Spain.

To study of the discriminative capacity of the BPFSC-11, a BPD group and an ADHD group were selected. Participants were included if: (1) they were between the ages of 9 and 18 years; (2) they did not present any learning disability; (3) there was no simultaneous diagnosis of BPD and ADHD; (4) there was no current diagnosis of schizophrenia, bipolar I disorder, or active substance dependence disorder; and (5) they were not suffering from any organic condition that could better explain the symptoms. In addition, to assess discriminant capacity between psychopathological characteristics and the inherent developmental characteristics of adolescents, a control group of healthy volunteers with no diagnosed psychiatric pathologies was included. Once the inclusion and exclusion criteria had been reviewed, all admitted subjects and their parents were invited to participate in the study, which was approved by the hospital’s Ethics Committee. Written informed consent for participation was obtained from all parents before inclusion in the study.

Table 1 presents the participants’ descriptive data. The total sample comprised 145 subjects, 62.1% female (*n* = 90). The mean age was 15.51 years (SD = 2.21), and 63.4% (*n* = 92) were mainly attending high school at the time of the study. Of these, 116 were outpatients being treated for BPD and ADHD (BPD and ADHD group; 40%) in the psychiatry department of a general university teaching hospital in Barcelona (Spain). And 29 were healthy adolescent volunteers recruited from school programs and were also tested at the hospital (20%; healthy control group).

**Table 1:** Sociodemographic and clinical characteristics of samples

	**BPD (*n* = 58)**	**ADHD (*n* = 58)**	**Control group (*n* = 29)**	**Post-hoc (Tukey’s test)**
**Sex (n, %)**				F = 26.289 (*p* < .001)
**Female**	49 (84.48)	18 (31.03)	23 (79.31)	BPD = Control > ADHD
**Male**	9 (15.52)	40 (68.97)	6 (20.69)	ADHD > BPD = Control
**Age (mean, SD)**	16.33 (1.66)	13.71 (1.83)	16.97 (1.74)	F = 47.234 (*p* < .001) BPD = Control > ADHD
**Academic level (n, %)**				*F* = 1.057 (*p* = *.35*)
**Primary school**	15 (25.9)	5 (8.6)	4 (13.8)	
**High school**	32 (55.2)	43 (74.1)	17 (58.6)	
**Vocational training**	5 (8.6)	4 (6.9)	1 (3.4)	
**University**	5 (8.6)	6 (10.4)	7 (24.1)	
	**BPD (*n* = 38)**	**ADHD (*n* = 12)**	**Control group (*n* = 21)**	
**KSADS/SCID-I (n, %)**				
**None**	19 (50)	7 (58.33)	21 (100)	F = 6.325 (*p* = .003)
**One disorder**	14 (36.84)	4 (33.33)	0 (0)	
**Two disorders**	3 (7.89)	0 (0)	0 (0)	BPD > Control
**Three or more disorders**	2 (5.3)	1 (8.3)	0 (0)	
**SCID-II (n, %)**				
**None**	3 (7.9)	10 (83.3)	21 (100)	F = 101.99 (*p* < .001)
**One or more PD (Other than BPD)**	35 (92.1)	2 (16.7)	0 (0)	BPD > ADHD = Control

Discrimination of Borderline Personality Disorder (BPD) and Attention-Deficit/Hyperactivity Disorder (ADHD) in Adolescents: Spanish Version of the Borderline Personality Features Scale for Children-11 Self-Report (BPFSC-11)

### Measures

The Borderline Personality Features Scale for Children-11 (BPFSC-11) [26] is an 11-item self-report instrument using a 5-point Likert response format, ranging from 1 (*not at all true*) to 5 (*always true*). It takes less than 10 min to administer. A cut-off point of 34 is considered optimal for the diagnostic identification of BPD (sensitivity = 0.74; specificity = 0.71) [26]. Concurrent validity was tested with the Childhood Interview for DSSM-IV Borderline Personality Disorder (CI-BPD) [29], yielding an Area Under the Curve (AUC) of 0.80 [30]. Due to its rapid administration, factorial structure, and optimal psychometric properties—especially reliability and construct validity— the original version of the BPFCS-11 has been considered an appropriate instrument for the clinical diagnosis of BPD and is also useful in epidemiological and follow-up studies [26]. With the authorization of the author, the BPFSC-11 was translated into Spanish by two independent, native-Spanish speakers with clinical expertise who were familiar both with the constructs being measured and with the target population. The translators reached a consensus on a common version, which was then blindly back-translated by a native English speaker and compared with the original scale. The above procedures followed the standard recommendations for adaptation of questionnaires [31].

The Spanish version of the Structured Clinical Interview for DSM-IV Axis II Disorders (SCID-II) [32] was used to assess the diagnosis of BPD and the other PDs according to Diagnostic and Statistical Manual of Mental Disorders IV and 5 (DSM-IV/DSM-5) criteria in subjects from 16 years of age. For younger individuals, personality traits were explored through a clinical interview during the assessment.

For the diagnosis of ADHD, the Schedule for Affective Disorders and Schizophrenia for School-Age Children-Present and Lifetime Version (K-SADS-PL) [33, 34] was used for adolescents under 16. In patients of 17 and 18 years, the Structured Clinical Interview for DSM-IV Axis I (SCID-I) [35] was used. Also, to complete this diagnosis of ADHD, the Spanish version of the Child Behavior Checklist (CBCL by Achenbach) was administered to parents to assess internalizing and externalizing symptoms in patients between 4 and 18 years old [36]. For all participants in the study, both K-SADS-PL and SCID-I interviews were used to assess current comorbid disorders.

## Procedure

This study is a preliminary analysis forming part of a general ongoing project to validate the Spanish v of the BPFSC-11. The adolescents had been diagnosed previously with BPD and ADHD by the respective programs in the hospital Psychiatry Department.

Psychopathological evaluation of BPD and ADHD participants was performed in three consecutive sessions by a psychiatrist (first session) and a clinical psychologist (second and third sessions), both with experience in the diagnosis of BPD and ADHD. Clinical and sociodemographic data were recorded in the first interview. During the second and third sessions, the K-SADS-PL or SCID-I interviews, the SCID-II, and the self-reported BPFSC-11 were administered by the same clinical psychologist. The material was administered in the same order for all participants.

The healthy control group was made up of adolescents with an absence of criteria for any psychiatric pathology, and who followed same diagnostic procedure as the previous groups.

### Data analysis

Comparisons between the BPD, ADHD and control groups were evaluated using two side T-tests, chi-square (χ^2^), or ANOVA tests, depending on the type of variable to be studied (Sociodemographic and comorbidity variables) and the groups to be compared. Since this work is part of a larger validation project, the present study included a preliminary analysis of the psychometric properties. Exploratory factorial analysis using principal components with oblique rotation was used with the whole sample to study the BPFSC-11 factorial structure. The Kaiser–Meyer–Olkin (KMO) test was used to analyze whether data was suitable for factor analyses and a value greater than 0.80 was considered adequate. Cronbach’s alpha was used to analyze BPFSC-11 reliability. Diagnostic performance was studied using the Receiver Operating Characteristic (ROC) curve. Two analyses were performed, one with the total sample (*n* = 145) and the other with the participants from the BPD and ADHD Groups (*n* = 116; clinical sample). Sensitivity and 1-specificity plot was used to select the cut-offs and the AUC was used to analyze the validity of the measurement. An AUC of 0.7 was considered an adequate result for validity [37]. Although a more appropriate approach based on odds of the studied disease has been proposed, in this preliminary study the approach used was the maximization of indices for both sensitivity and specificity [38]. Finally, between the groups (BPD, ADHD, and control groups) two-way ANOVA tests were used for the discriminative capacity of the BPFSC-11 total score and each of the scales. Statistical significance was established at *p* ≤ 0.05. Non-participants were excluded by missing data.

## Results

### Between-group comparison

Initially, between-group comparison for sociodemographic variables was performed. Significant differences between the groups were only observed for age (*p* < 0.001) and sex (*p* < 0.001). Post-hoc analyses indicated that the ADHD group was significantly younger than the healthy control group [ADHD group: 13.71 (1.83) vs. healthy control group: 16.97 (1.74), *p* < 0.001] and the BPD group [16.33 (1.66), *p* < 0.001] (see Table 1). Also, a significantly higher frequency of females was observed for the BPD and healthy control groups than for the ADHD group (*p* < 0.001) (see Table 1).

In relation to the SCID-II interview, and following the criteria for its administration by age, a sub-sample of clinical patients was used (see Table 1). The results obtained indicate the presence of differences in comorbidity with other PDs (*p* < 0.001) among clinical groups, with the highest being among BPD adolescents, and specifically those with avoidant personality disorder (AvPD) (39.5%), while in the ADHD group it was observed in just 2 patients (16.7%) with Cluster C. With regard to other comorbid disorders, no significant differences were found (*p* = 0.003), with anxiety disorder being the most prevalent among both clinical groups (13.2% BPD vs. 8.3% ADHD). Table 1 shows the results for each group of participants.

### Preliminary psychometric analysis

Analysis of the factor structure of the BPFSC-11 indicated a good fit index of the data (Kaiser–Meyer–Olkin = 0.88). Two factors were identified. Six of the 11 items of the BPFSC-11 (items 1, 4, 6, 7, 9, 10) were included in factor 1, and three were included in factor 2 (items 5, 8, and 11). Two items presented very similar loads in both factors (items 2 and 3). Factor 1 includes the items that refer to aspects of instability in emotions and interpersonal relationships, fear of abandonment and loneliness, guilt, extreme emotions, feelings of emptiness, and identity difficulties, and could therefore be called an emotional dysregulation factor. Factor 2 includes items that evaluate acting without thinking about the consequences, carelessness, cruelty, and lack of empathy, i.e., an impulsivity/recklessness factor. The emotional dysregulation factor explained 42.54% of the variance of the BPFSC-11 (eigenvalue = 4.68), and the impulsivity/recklessness factor explained only 9.90% (eigenvalue = 1.09). The correlation between the factors was *r* = 0.59. The factorial loads of the BPFSC-11 items are shown in Table 2.

**Table 2:** Factorial loadings for the 11 BPFSC Items included in the Factor Analysis (*n* = 145)

*Items BPFSC-11 Spanish Version*	*Factor*	
	***1***	***2***
**1**) I feel very lonely	**.84**	.31
**4**) I feel that there is something important missing about me, but I don’t know what it is	**.81**	.43
**10**) How I feel about myself changes a lot	**.78**	.40
**6**) People who were close to me have let me down	**.74**	.37
**7**) I go back and forth between different feelings, like being mad or sad or happy	**.73**	.52
**9**) I worry that people I care about will leave and not come back	**.63**	
**11**) Lots of times, my friends and I are really mean to each other		**.73**
**8**) I get into trouble because I do things without thinking	.39	**.71**
**5**) I'm careless with things that are important to me	.38	**.62**
**2**) I want to let some people know how much they've hurt me	.56	.52
**3**) My feelings are very strong. For instance, when I get mad, I get really really mad. When I get happy, I get really really happy	.46	.46
**Eigenvalue**	4.68	1.09
**% Variance**	42.54	9.91

Factor 1: Emotional dysregulation. Factor 2: Impulsivity/recklessness factor. Items loading on each factor are in boldface

The reliability of the total BPFSC-11 score based on the 11 items was α = 0.86 (mean α = 0.85) and did not increase when any item was eliminated. However, when the internal consistency of the BPFSC-11 was analyzed by factors, a somewhat higher alpha value for the emotional dysregulation factor (α = 0.89; 8 items) than for the impulsivity/recklessness factor (α = 0.59; 3 items) was obtained.

### Discriminative capacity of BPFSC-11 to distinguish between groups

ROC curve analyses were performed to study the cut-off point, sensitivity, and specificity of the BPFSC-11 and its factors. Two analyses were performed, the first with the total sample (*n* = 145) and the second with the participants from the BPD and ADHD groups (*n* = 116; clinical sample).

**Fig. 1:**
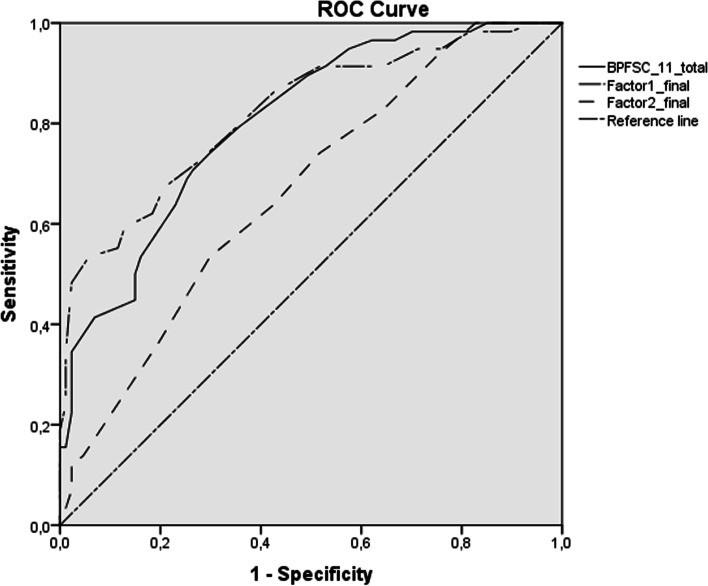
ROC curve comparing BPD group with the other participants (*n* = 145)

The first ROC curve analysis with the total sample indicated a good diagnostic fit for the BPFSC-11, with an AUC of 0.80 (95% CI [0.73, 0.87], *p* < 0.001) and an emotional dysregulation factor of 0.82 (95% CI [0.75, 0.89], *p* < 0.001). However, the AUC was lower than 0.70 for the impulsivity/recklessness factor (AUC = 0.66; 95% CI [0.58, 0.75], *p* = 0.001) than for factor 1. Sensitivity and specificity analysis indicated an adequate cut-off point of 30 to discriminate between BPD and all other participants for the BPFSC-11 (sensitivity = 0.74; specificity = 0.70), of 20 for factor 1 (sensitivity = 0.72; specificity = 0.72), and of 11 for the impulsivity/recklessness factor (sensitivity = 0.64; specificity = 0.57) (Fig. 1).

**Fig. 2:**
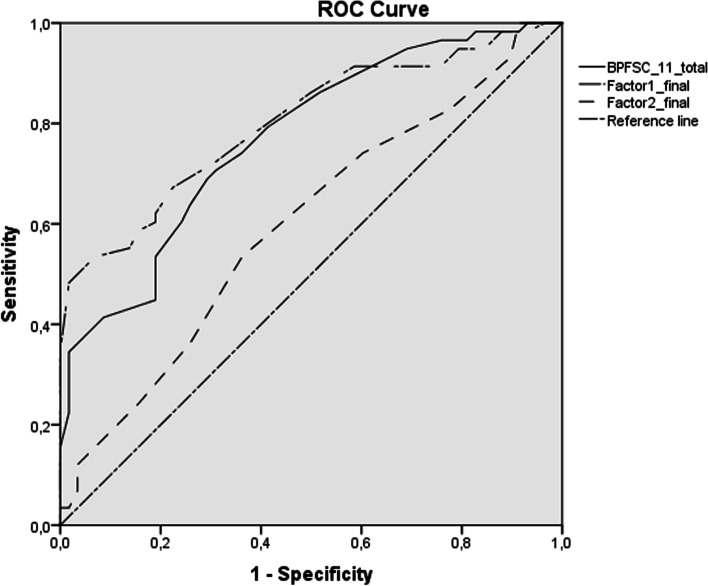
ROC curve comparing BPD and ADHD groups (*n* = 116)

Compared to the results of the first ROC curve analysis, the second analysis, which only included the clinical sample (*n* = 116), indicated a somewhat lower diagnostic fit compared with the results for the whole sample. Although it was optimal for the BPFSC-11 (AUC = 0.77, 95% CI [0.69, 0.85], *p* < 0.001) and for the emotional dysregulation factor (AUC = 0.80, CI 95%: 0.672–0.88; *p* < 0.001), the diagnostic fit of the impulsivity/recklessness factor was lower than that obtained in the previous analysis, without reaching a level of statistical significance (AUC = 0.60, 95% CI [0.50, 0.70], *p* > 0.05). Similarly, while for the same cut-off point, the sensitivity of the BPFSC-11 (0.74), emotional dysregulation factor (0.72) and impulsivity/recklessness factor (0.64) remained the same, the specificity value was lower for all three scores (BPFSC-11: 0.64, emotional dysregulation factor: 0.69 and impulsivity/recklessness factor: 0.52) (Fig. 2).

Finally, a one-factor ANOVA was performed to compare the BPFSC-11 scores among groups (Table 3). The results yielded significant differences both for the complete self-report and for each of the two factors obtained in the factor analysis [BPFSC-11: F _(2,142)_ = 33.25, *p* < 0.001; emotional dysregulation factor: F_(2,142)_ = 33.92, *p *< 0.001; impulsivity/recklessness factor: F_(2,142)_ = 15.10, *p* < 0.001]. Post-hoc tests indicated a significantly higher BPFSC-11 total mean score in the BPD group compared with the other two [BPD group: 35.83 (SD 7.98) vs. ADHD group: 27.74 (6.91) vs. healthy control group: 22.52 (8.34), all *ps* < 0.001], and for the emotional dysregulation factor [BPD group: 24.15 (6.35) vs. ADHD group: 17.26 (5.15) vs. healthy control group: 14.52 (5.78), all *ps* < 0.001]. However, despite the significantly higher mean impulsivity/recklessness factor in the BPD group compared with the healthy control group [11.67 (2.96) vs. 8.03 (3.16), *p* < 0.001], no significant differences were observed in the mean impulsivity/recklessness factor score between the BPD and ADHD groups [11.67 (2.96) vs. 10.59 (2.73), *p* > 0.05]. On the other hand, the BPFSC-11 total score [27.74 (6.91) *vs*. 22.52 (8.34), *p* < 0.01] and the score for the impulsivity/recklessness factor [10.59 (2.73) vs. 8.03 (3.16), *p* < 0.001] were significantly higher in the ADHD group compared with the healthy control group, but no differences between groups were observed for the emotional dysregulation factor score [17.26 (5.15) vs. 14.52 (5.78), *p* = 0.10].

**Table 3:** Mean (SD) of the total score of the complete BPFSC-11 Self-Report, Emotional Dysregulation Factor and Impulsivity/Recklessness Factor by group; differences by ANOVA analysis

	**BPD (*n* = 58)**	**ADHD (*n* = 58)**	**Control (*n* = 29)**	***F***_***(2, 142)***_
**BPFSC-11 Self Report**	35.8 (7.9)	27.7 (6.9)	22.5 (8.3)	33.246 (*p* < .001)
**Factor 1: Emotional dysregulation**	24.2 (6.4)	17.3 (5.1)	14.5 (5.8)	33.917 (*p* < .001)
**Factor 2: Impulsivity/recklessness**	11.7 (3.0)	10.6 (2.7)	8.0 (3.2)	15.099 (*p* < .001)

As significant differences between groups were observed for sex and age, a post-hoc analysis was performed to test the effect of these variables. The results showed a significant effect of sex (F_(3,140)_ = 9.09, *p* < 0.001), but not of age (F_(3,140)_ = 1.75, *p* = 0.16). Girls scored significantly higher than boys in the total BPFSC-11 [32.41 (9.49) versus 25.87 (7.09); t_(143)_ = 4.41; *p* < 0.001] and emotional dysregulation factor [25.45 (7.65) versus 19.27 (5.83); t_(143)_ = 5.15; *p* < 0.001], but no significant differences were observed between the two sexes for the impulsivity/recklessness factor [7.03 (2.72) versus 6.60 (2.17); t_(143)_ = 1.00; *p* = 0.32].

## Discussion

Although the diagnosis of BPD during adolescence is consistently recognized in current studies [7, 8], it remains controversial in clinical settings. The overlap between the clinical characteristics of BPD with other disorders, such as ADHD, can make BPD diagnoses unreliable. That is why early diagnosis and screening are necessary since they represent the first step to adequate monitoring and the implementation of specific treatments. In that sense, and coinciding with different authors, the non-identification of these disorders which emerge during periods of early development, while they may still be flexible and modifiable, may have negative repercussions when they evolve into a disruptive transition in adulthood [39]. While research has been stepped up in recent years, the scarcity of valid instruments for the detection and diagnosis of adolescents who are subsyndromal or have BPD hinders its evaluation and limits the accuracy of identification and discrimination procedures [39]. Knowledge of the discriminative capacity of the existing instruments is therefore necessary. The main objective of this preliminary study was to analyze the capacity of the BPFSC-11 to discriminate between BPD and ADHD in adolescents.

The results obtained in this study suggest that the BPFSC-11 total score seems to be a useful measure to discriminate between BPD and ADHD in adolescents. On the other hand, while typically a unidimensional factor structure is supported, our preliminary results also show a two-factor solution: emotional dysregulation and impulsivity/recklessness factors, which present differences in their capacities to discriminate between both disorders. These two results, which are discussed below, should be considered the main strengths of our study.

Regarding the first result, to our knowledge and to date, this is the first preliminary study of the Spanish version of the BPFSC-11 instrument for identifying discriminant capacity between BPD and ADHD in adolescents. Therefore, there have been no other published measures against which we can contrast our own results. However, these results are of major interest since, as pointed out by some authors [40], it is essential to identify the clinical conditions in adolescents at high risk of developing a severe and heterogeneous mental disorder such as BPD, and to differentiate this from other psychopathological disorders, thereby avoiding diagnostic errors with clinical implications in terms of early intervention programs. Having instruments available that can help regular clinical practice to distinguish between BPD and ADHD in adolescents and control their possible comorbidities with other psychiatric disorders, such as substance use disorder (SUD) and other affective disorders, could contribute to reducing poor psychopathological outcomes, as signaled in some recent studies [2]. As stated previously in the literature [25], by taking account of the developmental stage of adolescence described, the BPFSC-11 could help to improve this situation. Our results specifically encourage us to consider that this instrument can help to improve detection and discrimination between BPD and ADHD, thereby contributing to a greater understanding of their endophenotypes [2]. Future studies with this instrument should replicate these results.

In relation to the analysis of the factors’ discriminant capacity, although the sample size and the fact that this is a preliminary study mean that the outcomes must be taken with caution, the results obtained in relation to the two-factor solutions are interesting. The first emotional dysregulation factor of the BPFSC-11 is that which best discriminated between all groups, and especially between BPD and ADHD, and between BPD and healthy adolescents, but did not discriminate between ADHD and healthy adolescents. These results would be consistent with the idea that although emotional dysregulation is a common dimension of both disorders, it is more clearly related to BPD in adolescents as primary core pathology [25, 41, 42]. In contrast, these results may coincide with those of other studies which indicate that while a considerable number of ADHD patients present emotional dysregulation as a primary symptom, this does not occur in all of them [25]. Meanwhile, the lack of discriminant capacity observed between ADHD and healthy controls may be due to the fact that emotional instability in the former group could manifest itself clinically at a later stage, because during the earlier stages of ADHD, clinical features of a more behavioral nature are exhibited that are subsequently associated with that dysregulation. In other words, an earlier behavioral expression could well be the cause of an inadequate emotional response in later life [43], explaining the results found here.

Regarding the second factor, the results suggest that impulsivity/recklessness does not produce an adequate discrimination between BPD and ADHD, but it does between these two disorders and healthy adolescents. The literature indicates that impulsivity is a multifaceted construct which constitutes a characteristic psychopathological feature, a symptom and diagnostic criterion for both disorders [25]. However, it does not represent the same psychopathological description for both. While studies suggest that the type of impulsivity in BPD individuals is related to stress dependence, in ADHD patients it would be better defined in relation to a motor type, adopting more behavioral aspects such as impatience when waiting, talking and/or interrupting others [40]. Factor 2 of the Spanish version of the BPFCS-11 may, therefore, be more related to these behavioral characteristics and even more with antisocial behavior and lack of empathy, and less to those of BPD. It would therefore appear that our results reinforce the idea that, while BPD and ADHD share psychopathological traits, impulsivity/recklessness as evaluated in the BPFSC-11 self-report should be considered more of core pathology of ADHD. However, taking into account the small number of items that make up this factor, which explain a small variance, and the aspects that they evaluate, our results would seem to suggest that they are more typical of ADHD than of BPD, differentiating it even more clearly from the control group adolescents. On the other hand, and although some authors have argued a greater association between the male sex and impulsivity, in our study this factor does not seem to discriminate in relation to sex either [26]. Therefore, the results obtained should be interpreted with caution and in the expectation of obtaining more robust outcomes in future studies. One option to consider might be to increase the number of items included with a view to increasing the weighting of a factor that may be relevant for discriminating between BPD and ADHD in adolescents.

Finally, and in relation both factors, while we agree with studies such as those by Chanen et al. (2016) which indicate the importance of identifying adolescents with specific traits or subthreshold symptoms as necessary for on-time intervention, [24, 44], future studies should also analyze their possible association with some of the maladaptive traits of Criterion B of the hybrid model of personality disorders of the DSM-5 and DSM-5-TR. Specifically, emotional dysregulation could be related to characteristics of negative affectivity or neuroticism, as well as impulsiveness/recklessness with lack of inhibition [45]. Both dimensions could be related to these BPD criteria, which could be more interesting to study in the psychopathological discrimination of adolescence. Therefore, recent studies such as Thomson et al. (2022) suggest the need to have available instruments that examine clinical usefulness and not just in detecting of personality disorders according to purely categorical conceptualizations, but which also evaluate their capacity to catch them in accordance with new dimensional constructs such as impairment in personality functioning in adolescents [46].

This study has some limitations but also some strengths. The first limitation is that the Spanish validation of the BPFSC-11 has not yet been finalized. However, its preliminary psychometric results (internal consistency and reliability, sensitivity and specificity) were similar to the original version of the BPFSC-11 [26]. The second limitation is the small size of the samples. Although the study was initially designed to be performed over a more extended time, the recruitment of participants, especially for the control group, was affected by the Covid-19 pandemic, meaning that the number was limited. Therefore, given the total lockdown, we decided only to include the participants recruited up to February. However, in future studies the number of participants should be higher, and the healthy control group increased to be able to confirm the results obtained more confidently and to understand the similarities or differences in the clinical risk profiles. Therefore, the results related to the two-factor solutions, although interesting, must be taken with caution and confirmed in future studies with higher sample sizes. The third limitation relates to the evaluation made using the SCID-II interview in clinical samples. While this has been used successfully in in studies with adolescents, the age of many of the participants in this study made its administration impossible, especially for the ADHD group where the participants were the youngest. Future studies should include other, more appropriate methods developed specifically to assess adolescent PD such as the CI-BPD, which could be interesting for the most objective way of studying personality structure, as well as a larger study of comorbidities. Finally, as a fourth limitation, a significant effect of sex was observed in the study, with an overrepresentation of girls in the BPD group. Future studies should study more balanced samples in terms of gender distribution.

## Conclusions

To conclude, the results of this study seem to support the idea that the BPFSC-11 can be a useful instrument for the identification of adolescent BPD. Also, and related to disorders with overlapping symptoms, the emotional dysregulation factor seem useful for discriminating between adolescents with BPD and those with ADHD. These results should be considered of interest since they can help to improve the evaluation of this pathology in this population, and also support the necessary discrimination from other disorders with similar clinical features, thereby improving their diagnosis. Consequently, this could benefit the subsequent implementation of psychotherapeutic interventions in adolescents with these pathologies and improve their situations and futures.

## Data Availability

The data that support the findings of this study are available on reasonable request from the corresponding author.
